# Antimicrobial resistance in Libya: 1970–2011

**DOI:** 10.3402/ljm.v8i0.20567

**Published:** 2013-03-27

**Authors:** Khalifa Sifaw Ghenghesh, Amal Rahouma, Khaled Tawil, Abdulaziz Zorgani, Ezzedin Franka

**Affiliations:** 1 Department of Microbiology and Immunology, Faculty of Medicine, University of Tripoli, Tripoli, Libya; 2 Department of Family and Community Medicine, Faculty of Medicine, University of Tripoli, Tripoli, Libya

**Keywords:** antibiotic resistance, enteric bacteria, methicillin-resistant *Staphylococcus aureus*, multidrug-resistant tuberculosis, Libya

## Abstract

Resistance to antimicrobial agents is a major health problem that affects the whole world. Providing information on the past state of antimicrobial resistance in Libya may assist the health authorities in addressing the problem more effectively in the future. Information was obtained mainly from Highwire Press (including PubMed) search for the period 1970–2011 using the terms ‘antibiotic resistance in Libya’, ‘antimicrobial resistance in Libya’, ‘tuberculosis in Libya’, and ‘primary and acquired resistance in Libya’ in title and abstract. From 1970 to 2011 little data was available on antimicrobial resistance in Libya due to lack of surveillance and few published studies. Available data shows high resistance rates for *Salmonella* species in the late 1970s and has remained high to the present day. High prevalence rates (54–68%) of methicillin-resistant *Staphylococcus aureus* (MRSA) were reported in the last decade among *S. aureus* from patients with burns and surgical wound infections. No reports were found of vancomycin-resistant *S. aureus* (VRSA) or vancomycin-intermediate-resistant *S. aureus* (VISA) using standard methods from Libya up to the end of 2011. Reported rates of primary (i.e. new cases) and acquired (i.e. retreatment cases) multidrug-resistant tuberculosis (MDR-TB) from the eastern region of Libya in 1971 were 16.6 and 33.3% and in 1976 were 8.6 and 14.7%, in western regions in 1984–1986 were 11 and 21.5% and in the whole country in 2011 were estimated at 3.4 and 29%, respectively. The problem of antibiotic resistance is very serious in Libya. The health authorities in particular and society in general should address this problem urgently. Establishing monitoring systems based on the routine testing of antimicrobial sensitivity and education of healthcare workers, pharmacists, and the community on the health risks associated with the problem and benefits of prudent use of antimicrobials are some steps that can be taken to tackle the problem in the future.

Resistance to antimicrobial drugs is a major health problem that affects the whole world. The problem is still worse in developing countries where lack of antimicrobial-resistance surveys and control policies are the norm. In Libya, misuse of antimicrobial agents by the public is widespread. As in many developing countries antimicrobials can be purchased from pharmacies without prescription in Libya. A study carried out on self-medication with antibiotics in the ambulatory care setting within eight countries of the Euro-Mediterranean region between 2004–2005 found 19.1% (range ≤0.1% in Cyprus to 37% in Lebanon) of total interviewed healthy individuals admitting self-medication and in Libya the percentage was 24% (1). The study also found that nearly 50% of interviewed Libyans indicated that they would take antibiotics for their own use without a prescription if they believe that they needed to. Furthermore, antibiotics can easily be obtained in Libya on demand from, usually but not always, relatives, friends, and others employed in government medical stores and hospitals (KS Ghenghesh, unpublished data).

In the past, the state of health in Libya was not good particularly in the few years prior to the 17 February 2011 uprising. The reasons for this include, among others, lack of good administration, resources, maintenance of hospitals, well-trained healthcare workers, and surveillance of different diseases and support of biomedical research. The poor state of health undoubtedly aggravated the problem of antimicrobial resistance in the country. However, providing information on the past state of antimicrobial resistance in Libya may assist the health authorities in addressing the problem more effectively in the future.

The objective of the present work was to review the published data and available reports on antimicrobial resistance in Libya, covering mainly three important topics of antimicrobial resistance in enteric bacteria, methicillin-resistant *Staphylococcus aureus* (MRSA), and multidrug-resistant tuberculosis (MDR-TB) from 1970 to 2011. The information presented in this review was obtained from Highwire Press (including PubMed) search for the period 1970–2011 using the terms ‘antibiotic resistance in Libya’, ‘antimicrobial resistance in Libya’, ‘tuberculosis in Libya’, and ‘primary and acquired resistance in Libya’ in title and abstract. Additional data were also obtained from a Google search using the aforementioned terms. Furthermore, papers published in local biomedical journals, and when available, abstracts presented in local and international meetings on the subject were included.

## Enteric bacteria

These include members of the family *Enterobacteria*ceae mainly *Salmonella* and *Shigella* species from stool samples of diarrheic patients and *Escherichia coli* from cases of urinary tract infections (UTI). Other enteric bacteria isolated less frequently from UTI include *Klebsiella*, *Enterobacter*, and *Proteus* species.

## *Salmonella* and *Shigella*

Resistance rates for *Salmonella* varied tremendously among the different commonly used antibiotics. High resistance rates for ampicillin were reported in 1979–2008 from Tripoli (Table 1).

**Table 1:** Antimicrobial resistance of *Salmonella* isolated from diarrheic fecal specimens from 1979 to 2008 in different Libyan cities

		% resistant to:								
			
Year/city	No. tested	Amp	AMC	CEF	C	Gen	NA	Cip/Nor	TMX	References
1979/Tripoli	244^a^	89	NT	NT	79	17	12	NT	NT	(2)
1979/Tripoli	238^b^	29	NT	NT	89	14	17	NT	NT	(2)
1992–1993/Tripoli	21	52	NT	43	52	43	0.0	0.0	48	(3)
1993/Benghazi	157	70	58.5	NT	66.2	64.3	NT	NT	67.5	(4)
2000–2001/Zliten	23	100	96	NT	96	78	4	0.0	4	(5)
2008/Tripoli	19	47	5	57.8	5	NT	84	63	21	(6)

Amp, ampicillin; AMC, amoxicillin-clavulanic acid; CEF, cephaloridine; C, chloramphenicol; Gen, gentamicin; NA, nalidixic acid; Cip/Nor, ciprofloxacin/norfloxacin; TMX, trimethoprim-sulfamethoxazole; NT, not tested.

a *S. wein* isolates.

b *S. muenchen* isolates.

In the mid-1980s, Sood et al. in Benghazi reported on 38 children with acute gastroenteritis due to *Salmonella* treated with gentamicin, to which the organisms were sensitive in-vitro (7). Most (30 out 38) of the stool cultures from the children remained positive after treatment. The authors concluded that antibiotics have no role to play in the treatment of pediatric *Salmonella*-associated acute gastroenteritis.

During the period 1990–1999, more than 40% of *Salmonella* and *Shigella* from children with diarrhea were resistant to ampicillin and trimethoprim-sulfamethoxazole (3, 8). Similar findings were reported for *Salmonella* from Benghazi (4). A study carried out in Zliten city between 2001 and 2002 reported multidrug resistance (MDR, resistance to three or more antibiotics) of more than 75% among *Salmonella* species isolated from diarrheic children (9). Recently, Rahouma et al. reported that 63% (12/19) of *Salmonella* species isolated from diarrheic children in Tripoli were resistant to ciprofloxacin (6). Their finding is a serious health problem as fluoroquinolones are indicated for the treatment of severe salmonellosis in adults (10).

### Escherichia coli

*Escherichia coli* is the predominant causative agent of acute UTI worldwide. A study from Benghazi in the early 1980s reported 22% resistance to ampicillin among *E. coli* from UTI (11). The rate of resistance to ampicillin increased nearly three-fold from 1990 to 1999. High rates of resistance to trimethodprim-sulfamethoxazole were also observed during the same period for *E. coli* from UTI in Tripoli and Benghazi. However, studies carried out between 2002 and 2008 reported lower resistance rates to ampicillin and trimethoprim-sulfamethoxazole and an increase in resistance rates to fluoroquinolones.

Ciprofloxacin and other fluoroquinolones are drugs of choice in the treatment of UTI in adults, particularly in areas where resistance to ampicillin and trimethoprim-sulfamethoxazole among uropathogens are high (12). These drugs became available in pharmacies in Libya (mainly in Tripoli and Benghazi) in the late 1990s (KS Ghenghesh, personal observation). The use of ciprofloxacin in recent years may lead to a reduction in the use of ampicillin and trimethoprim-sulfamethoxazole for the treatment of UTI in Libya. This may in turn have resulted in the increased resistance to the former antibiotic and reduced resistance to the latter drugs. Table 2 shows the antimicrobial-resistance profile of *E. coli* isolated in the period 1981–2008 from acute UTI in different Libyan cities.

**Table 2:** *Escherichia coli* isolated during 1981–2008 from urinary tract infections in different Libyan cities

		% resistant							
			
Year/city	No. tested	Amp	C	Gen	NA	Cip	NF	TMX	References
1981/Benghazi	38	22	NT	0.0	0.0	NT	0.0	NT	(11)
1993–1995/Tripoli	31	97	29	6	0.0	NT	13	55	(13)
1994–1995/Tripoli	534	74	29	7	11	NT	25	45	(14)
1996/Benghazi	148	75	45	18	10	NT	7	81	(15)
2002–2005/Sirte	1265	49	23	9	28	2	7	36	(16)
2005–2006/Tripoli	29	59	21	10	28	14	NT	24	(17)
2006–2008/Benghazi	105	57	14	7	23	17	0.0	31	(18)

Amp, ampicillin; C, chloramphenicol; Gen, gentamicin; NA, nalidixic acid; Cip, ciprofloxacin; NF, nitrofurantoin; TMX, trimethoprim-sulfamethoxazole; NT, not tested.

*Escherichia coli* strains are part of the normal gut flora. However, some types known as diarrheagenic *E. coli* (DEC) are important intestinal pathogens that cause a variety of gastrointestinal diseases particularly among children in developing countries (19). A study examined 155 *E. coli* strains isolated from diarrheic children in Tripoli between September 1992 and August 1993 for their susceptibility to antibiotics. Of the strains examined, 79% were resistant to ampicillin, 23% to cephalexin, 12% to chloramphenicol, 26.5% to kanamycin, 2% to NA, 65% to tetracycline, and 50% to trimethoprim-sulfamethoxazole (20).

Recently, a study tested 21 strains of DEC (10 enteroaggregative *E. coli*, 3 enteropathogenic *E. coli*, and 8 enterotoxigenic *E. coli*) isolated from Libyan children with diarrhea in Zliten and Alkhoumes cities identified by PCR for their susceptibility to antibiotics (21). Of the DEC strains tested, 81% were resistant to ampicillin, 57.1% to amoxicillin-clavulanic acid, 42.9% to chloramphenicol, 9.5% to Nalidixic acid, and 61.9% to trimethoprim-sulfamethoxazole. All strains were susceptible to ceftriaxone, ciprofloxacin, and imipenem.

### Other enteric bacteria

Ghenghesh et al. examined 215 strains of enterobacteria isolated from human feces, urine, foods and waters during 1991–1993 in Tripoli and represent the following genera: *Escherichia*, *Citrobacter*, *Serratia*, *Kluyvera*, *Klebsiella*, *Enterobacter*, *Morganella*, *Proteus*, *Providencia*, as well as *Salmonella* and *Shigella* (22). Of the organisms examined, 57.7% were resistant to ampicillin, 25.6% to chloramphenicol, 6% to gentamicin, 29.3% to kanamycin, 28.4% to trimethoprim-sulfamethoxazole, and 65.6% to tetracycline.

## Extended-spectrum β-lactamases in *Escherichia coli* and *Klebsiella pneumoniae*

Extended-spectrum β-lactamases (ESBLs) are enzymes produced by bacteria, mostly *E. coli* and *Klebsiella* species, rendering them resistant to cephalosporins including cefotaxime, cefuroxime, and ceftazidime. These enzymes were first reported in the mid-1980s mainly in hospitals. A study carried out in 2002 reported ESBL in 8.6% of 383 *E. coli* and in 15.3% of 209 *K. pneumoniae* isolated from different clinical specimens submitted by Trauma and Surgery Departments in Tripoli Central Hospital (23). The study also found that production of ESBL is significantly associated with *E. coli* and *K. pneumoniae* from inpatients compared with outpatients (*P*< 0.000001). Production of ESBL in *E. coli* and *Klebsiella* species from Tripoli and Benghazi cities is shown Table 3.

**Table 3:** Extended spectrum β-lactamases in *Escherichia coli* and *Klebsiella* species from Libya

		*E. coli*		*Klebsiella* species		
				
Year/city	Source	No. tested	% ESBL	No. tested	% ESBL	References
2002/Tripoli	Clinical specimens	383	8.6	209	15.3	(23)
2005–2006/Tripoli	Urine	48	4	NT	NT	(17)
2000–2007/Tripoli	BICU^a^	NT	NT	32	47	(24)
2006–2008/Benghazi	Urine	105	1.9	42	2.4	(18)

NT, not tested.

a Blood specimens from patients with microbiological evidence of septicemia admitted to the burn intensive care unit.

## Methicillin-resistant *Staphylococcus aureus* (MRSA), vancomycin-resistant *S. aureus* (VRSA), vancomycin-intermediate *S. aureus* (VISA), and heterogeneous VISA (hVISA)

*Staphylococcus aureus* is well established as an important cause of hospital- and community-acquired infections. In addition, *S. aureus* isolates that are resistant to methicillin (MRSA) are resistant to all β-lactam drugs, and often to aminoglycosides, which makes them difficult to treat, particularly in hospitals (25, 26).

The first report of MRSA from Libya was by Goda in 1976 (27). He examined 16 *S. aureus* strains isolated from mastitis in cows in Benghazi during 1971–1972 for their susceptibility to six antibiotics including methicillin and oxacillin. Goda found that 25% (4/16) and 19% (3/16) were resistant to oxacillin and methicillin, respectively. Of the 16 *S. aureus* strains examined, 11 gave conflicting results between methicillin and oxacillin. However, Goda did not use control organisms nor did he compare the zones of inhibition obtained to the criteria set by the Food and Drug Administration at the time of the study as required by the standard method of Bauer and Kirby (28) used to determine the susceptibility of bacteria to antibiotics. This may explain the conflicting results obtained by the author and in turn render his results questionable.

Until the early 1990s, data regarding *S. aureus* and MRSA infections in Libya were lacking. However, an outbreak of MRSA occurred in 1981 in the Zurich-area University Hospital, Switzerland was related to a patient with multiple injuries from Libya (29). This indicates that MRSA was present in Libya since that time but not reported. In recent years, the highest prevalence rate (54–68%) of MRSA from Libya was found among *S. aureus* from patients with burns and surgical wound infections (30, 31). Isolation of MRSA from different sources in Libyan cities in the period 1995–2008 is shown in Table 4.

**Table 4:** Isolation of methicillin-resistant *Staphylococcus aureus* (MRSA) from different sources in Libyan cities in the period 1995–2009

Year/city	Source	No. tested	% MRSA	References
1995–1996/Tripoli	Different food samples	23	<1	(32)
1995–1996/Tripoli	Different clinical specimens	40	<1	(32)
1998/Tripoli	Used banknotes	50	4	(33)
1998–2001/Tripoli	Neonatal SCU^a^	25	12	(34)
2001–2002/Tripoli	Ice cream	60	<1	(35)
2004/Tripoli	DVDs	35	11	(36)
2005–2006/Tripoli	UTI	24	21	(17)
2007/Benghazi	Different clinical specimens	200	31	(30)
2007/Tripoli	Burn patients	120	54	(31)
2000–2007/Tripoli	Burn ICU^b^	174	55	(24)
2008/Tripoli	Healthcare workers	643	37	(37)
2009/Sabrata	Healthcare workers	43	11	(38)

a Blood specimens from babies attended special care unit with a clinical diagnosis of neonatal sepsis.

b Blood specimens from patients with microbiological evidence of septicemia admitted to the burn intensive care unit.

From this table, it can be noticed that the reported rates of MRSA from clinical sources were on the rise as years go by. The rise can be due to several factors that include an increase in the β-lactam drugs consumption in the country and better detection and reporting of MRSA. The lack of surveying studies in the country makes it difficult to pinpoint the causes associated with the increase in MRSA. However, in the last decade of this study, the prevalence of MRSA from clinical specimens in Libya was within the range reported for the Mediterranean region, which constitutes a high prevalence area for MRSA (39).

Conversely, there are no reports of MRSA from food sources in Libya. Of 60 *S. aureus* strains isolated from ice cream samples during January 2001 and October 2002 in Tripoli, none (0.0%) were MRSA (35). More studies are needed in the future to determine the prevalence of MRSA from different foods in the country.

In 1996, the first VISA was reported from a patient in Japan with MRSA pneumonia that was not responding to vancomycin treatment (40). VRSA are defined as those isolates having minimum inhibitory concentrations (MICs) ≥16 µg/mL, VISA with MICs between 4 µg/mL and 8 µg/mL, and hVISA strains that appear susceptible to vancomycin, but contain a subpopulation of cells with reduced susceptibility to vancomycin (MICs ≥4 µg/mL) (41). No VRSA, VISA, and hVISA using standard methods (i.e. CLSI) were reported from Libya up to the end of 2011. Recently, Ahmed et al. examined 170 isolates of *S. aureus* previously identified as MRSA by microbiology laboratories of three hospitals in Tripoli (42). They found only 51% (86/170) of them being MRSA by the cefoxitin disk diffusion method and PBP2a. They also reported none of the previously identified VRSA to be resistant to vancomycin by the E-test. Misidentification of MRSA and VRSA could be more common in a developing country where resources as well as skilled laboratory personnel are limited. Only 15 cases of VRSA have been reported worldwide (one from India, one from Iran, and 13 from the USA) since the first report in 2002 (43, 44). Physicians in Libya should be alert to any reports of VRSA and/or VISA and should interpret them very carefully.

## Tuberculosis (TB) and other respiratory tract infections

Tuberculosis (TB) caused by the bacterium *Mycobacterium tuberculosis* is a serious health problem in Libya. The WHO estimated TB incidence rate in Libya for the period 1990–2010 at 40 per 100.000 (45). The recommendation of the National Institute for Health and Clinical Excellence (NICE) for vaccination and screening in England and Wales considers countries with the above-mentioned incidence rate or greater to have a high incidence of TB (46).

Anti-tuberculosis agents are classified mainly into first- and second-line drugs. First-line agents are the most effective anti-TB drugs that include isoniazid, rifampicin, ethambutol, and pyrazinamide. In the past, streptomycin was considered a first-line drug; however, a worldwide increase in the frequency of resistance to this agent has made it less useful (47). Second-line anti-TB drugs have limited bactericidal capacity and are less effective and more toxic and much more expensive compared with first-line agents (48, 49). These agents include injectable aminoglycosides (kanamycin, amikacin, capreomycin), ethionamide, cycloserine, para-aminosalicylic acid, and fluoroquinolones (levofloxacin, moxifloxacin, ofloxacin, ciprofloxacin).

Multidrug-resistant tuberculosis (MDR-TB) is defined as tuberculosis caused by *M. tuberculosis* strains resistant to at least isoniazid and rifampicin. MDR-TB can be either a ‘new case’ (primary resistance) defined as a newly registered episode of TB in a patient with no prior anti-tuberculosis treatment, or a ‘previously treated case or retreatment case’ (acquired resistance) defined as a newly registered episode of TB in patient treated for TB for 1 month or more (50). Extensively drug-resistant TB (XDR-TB) is resistant to the same drugs as MDR-TB, plus any fluoroquinolone and at least one second-line aminoglycoside. MDR-TB and XDR-TB cannot be treated with the standard 6 months course of first-line agents and can take up to 2 years or more to treat with second-line drugs (49).

In the past, Libya never reported data on MDR-TB to the WHO. Also, there is no information on the effect of HIV on the prevalence of MDR-TB in the country. Data reported by the WHO on MDR-TB in Libya from 2002 to 2011 are estimates (49, 51, 52). However, few studies on the rate of MDR-TB in Libya have been reported in the biomedical literature. Khalil and Sathianathan examined the records of 771 Libyans with culture-positive cases of newly diagnosed pulmonary tuberculosis and 789 cases of both Libyans and non-Libyans with positive cultures during treatment on the Regional Tuberculosis and Chest Diseases Centre in Benghazi over a period of approximately 5 years (June 1971-August 1976) (53). They reported that the primary (new cases) and acquired resistance (retreatment cases) to streptomycin, isoniazid, and para-aminosalicylic acid in 1971 were 16.6 and 33.3%, and in 1976 were 8.6 and 14.7%, respectively. The authors indicated that the decline in both new and retreatment cases was mainly due to the introduction of anti-tuberculosis legislation in Libya in 1973. Another study from the western region of Libya examined a total of 598 and 246 strains of *M. tuberculosis* isolated during 1984–1986 for new and retreatment MDR-TB, respectively. MDR-TB was found to be 11% among new cases and 21.5% in retreatment cases (54).

In general, it can be observed from available data that new MDR-TB cases decreased significantly in the first decade of 21st century compared with the early 1970s. On the other hand, retreatment MDR-TB cases remained high (21–39%) during 1971–2011. Improvement in the standard of living and health services compared with that in 20th century pre-1970s era may represent some of the reasons for the decline in cases of new MDR-TB. However, rates of previously treated MDR-TB remaining high for several years have been reported from many countries worldwide (51). This is due to several factors that include duration of treatment that lasts for months which results in high rates of new case MDR-TB patients defaulting treatment. Rates of new and retreatment cases of MDR-TB in Libya from 1971 to 2011 are shown in Table 5.

**Table 5:** Rates of new and retreatment cases of multidrug-resistant tuberculosis (MDR-TB) in Libya

	% MDR-TB		
		
Year/region	New cases	Retreatment cases	References
1971–1976/ER	13.5 (8.6–16.6)	27.5 (14.7–33.3)	(53)
1984–1986/NWR	11 (4–20)	21.5 (12–34)	(54)
2002–2007/Libya^a^	2.6 (0.4–14.4)	38.7 (9.7–77.3)	(51)
2008/Libya^a^	2.9 (0.0–8.0)	35.4 (0.0–75.1)	(49)
2011/Libya^a^	3.4 (0.1–11)	29 (2.6–56)	(52)

ER, eastern region of Libya; NWR, north western region of Libya.

a World Health Organization estimates.

### Other respiratory infections

Data regarding antimicrobial resistance among other bacteria associated with respiratory tract infections and meningitis are scarce. A study from Benghazi examined 18 strains of *H. influenzae*, 17 strains *Streptococcus pneumoniae* and one strain of *N. meningitidis* isolated from pediatric cases of acute purulent meningitis from April 1994 to May 1995 (55). Resistance rates of 23 and 11% were found in *H. influenzae*, and 12 and 0% in *S. pneumoniae* to ampicillin and chloramphenicol, respectively. Furthermore, the investigators found all tested strains to be susceptible to ceftriaxone and 18% of *Str. pneumoniae* strains resistant to penicillin.

## Conclusion

In conclusion, the problem of antibiotic resistance is very serious in Libya and appears to be on the rise. High resistance rates were observed among enteric bacteria against commonly used drugs (i.e. ampicillin, trimethoprim-sulfamethoxazole, cephalosporins). In addition, MRSA was common in Libyan hospitals in the last decade, frequently detected in healthcare workers and patients. Furthermore, MDR-TB remains an impediment particularly among retreatment cases in the country. The health authorities in particular and society in general should address the problem of antimicrobial resistance urgently. Establishing monitoring systems based on routine testing of antimicrobial sensitivity and education of healthcare workers, pharmacists, and the community on the health risks associated with the problem and benefits of prudent use of antimicrobials are some of the steps that can be taken to tackle the problem in the future.

## Recommendations

Antimicrobial resistance is a threat to health associated with a heavy financial burden (56). The Libyan health authorities urgently need to allocate resources for surveillance of antimicrobial resistance using WHO guidelines and starting with hospital-acquired infections caused by MRSA, Gram-negative bacilli, and MDR-TB. Also, surveillance of antimicrobial use is needed. Information from both surveillance programs will provide data required to direct policy on the prudent use of antimicrobials and to apprize and evaluate resistance containment interventions at local and national levels (56).

In addition, reducing the impact of hospital-acquired (nosocomial) infection in our hospitals is urgently required. Such action will most likely reduce antimicrobial use in the hospital setting and may lead to a reduction in high rates of antimicrobial resistance reported from hospitals in Libya. Programs dealing with control of nosocomial infections in the country should be strengthened and updated regularly.

A major component of future policies for prevention and control of antimicrobial resistance in Libya should be education of healthcare workers, pharmacists, students, and the general public. Examples of classroom approaches for teaching the dynamics of antibiotic resistance that can be adopted and used and articles that may encourage teachers to understand that antibiotic resistance is a serious health problem and should be discussed with students in biology classes in our high schools and universities are available (57, 58). There are many international agencies (particularly the WHO), scientific societies, and other institutions that provide excellent and accurate educational resources on the subject for free (see supplemental box). Such agencies can be consulted and their educational resources should be used as guidelines. In addition, local scientific and culture societies, sport clubs, mosques, schools, universities, welfare and correctional centers, and the media should be involved in such programs. The crisis of antimicrobial resistance in Libya has reached a stage that requires the Ministries of Health, Education, and Higher Education and Scientific research to join forces in addressing this issue.
